# Host-virus molecular arms race: RNAi-mediated antiviral defense and viral suppressor of RNAi

**DOI:** 10.1016/j.cellin.2025.100276

**Published:** 2025-08-27

**Authors:** Bowen Zhang, Xi Zhou, Yujie Ren

**Affiliations:** a State Key Laboratory of Virology and Biosafety, Wuhan Institute of Virology, Chinese Academy of Sciences, Wuhan 430071, Hubei, China; b University of Chinese Academy of Sciences, Beijing 100049, China

**Keywords:** RNAi, VSR, Antiviral immunity, Drug, Vaccine

## Abstract

RNA interference (RNAi) is a highly conserved post-transcriptional gene silencing (PTGS) mechanism widely presented in eukaryotes. During viral infection, double-stranded RNA viral replicative intermediate (vRI-dsRNA) derived from the viral genome is recognized and processed by Dicer to generate small interfering RNA (siRNA). The viral siRNA (vsiRNA) is subsequently loaded into the RNA-induced silencing complex (RISC), which targets and degrades viral RNAs to achieve antiviral immune response. During long-term evolution, viruses have evolved to counteract RNAi by encoding viral suppressors of RNAi (VSRs) through various strategies, thereby evading the immune clearance. Here we review how VSRs function as immune evasion factors against antiviral RNAi, along with their evolutionary significance in shaping both viral adaptation and host-pathogen co-evolution. We also discuss recent advancements and unresolved controversies regarding RNAi-mediated antiviral immunity in mammals. Finally, we provide a comprehensive analysis of emerging therapeutic strategies and vaccine designs that leverage the RNAi-VSR interaction mechanisms, while addressing their potential prospects and challenges in clinical translation.

## 1 Introduction

RNA interference (RNAi) is a highly conserved gene expression regulatory mechanism widely found in eukaryotes (Fire et al., 1998). RNAi primarily achieves post-transcriptional gene silencing (PTGS) through non-coding small RNAs that are processed by Dicer and loaded into Argonaute (AGO) proteins. RNAi has been reported to participate in transcriptional-level gene silencing (TGS) through the RNA-directed DNA methylation in plants (Pontier et al., 2012; Sijen et al., 2001). Besides, RNAi also plays roles in regulating histone modifications, such as H3 Lys9 methylation, leading to the silencing of specific genes within heterochromatin and driving phenomena like position effect variegation (PEV) in *Drosophila* (Pal-Bhadra & Chikka, 2013). Recent studies have also reported TGS phenomena in mammals, where promoter-directed siRNAs regulate methylation modifications of target sequences to suppress endogenous gene transcription (Morris et al., 2004). Non-coding small RNAs such as microRNAs (miRNAs) generated in the RNAi pathway can load into the RISC to degrade mRNA or inhibit translation via perfect or imperfect base pairing. RNAi plays a vital role in maintaining physiological homeostasis and defending against exogenous pathogen invasion.

## 2 RNAi acts as an antiviral defense pathway

### 2.1 Mechanism of antiviral RNAi

Accumulating evidence in recent decades demonstrates that RNAi plays a significant antiviral role in eukaryotes. The double-stranded RNA viral replicative intermediates (vRI-dsRNAs) are recognized as pathogen-associated molecular patterns (PAMPs) by pattern recognition receptors (PRRs), including the RNase III enzyme Dicer, producing abundant virus-derived siRNAs (vsiRNAs), which are approximately 21–23 nucleotides (nt) in the length with 2-nt 3′ overhangs (Li & Ding, 2022). Dicer, the key initiator endoribonuclease in RNAi, consists of five critical domains: the N-terminal helicase (HEL) domain, domain of unknown function (DUF283), PIWI-AGO-Zwille (PAZ) domain, two tandem RNase III domains, and dsRNA-binding domains (dsRBDs). These domains work together to enable Dicer to cleave vRI-dsRNAs into small interfering RNAs (siRNAs): The dsRBD recognizes and nonspecifically binds to dsRNA, stabilizing the interaction between Dicer and dsRNA. The PAZ domain recognizes the terminus of the dsRNA substrate; the two RNaseIII domains approximate to form one catalytic center and cleave the opposing strands of dsRNA in an offset manner, resulting in a characteristic 2-nt 3’ overhang (Liu & Paroo, 2010). Notably, the helicase domain of mammalian Dicer proteins lacks helicase activity despite retaining ATPase activity (Zapletal et al., 2022; Zhang et al., 2002). Several Dicer co-factors are involved in the entire process of siRNA biogenesis and subsequent loading into the RISC, such as Loqs-PD and R2D2 for *Drosophila* Dicer-2 (Dcr-2) or PACT and TRBP for mammalian Dicer (Gredell et al., 2010; Lee et al., 2006; Liu et al., 2003; Saito et al., 2005; Tants et al., 2017; Tomari et al., 2004). Furthermore, Ars2 directly interacts with *Drosophila* Dcr-2 to enhance its processing efficiency toward dsRNA (Sabin et al., 2009). The siRNAs are then incorporated into the RISC, where the core component Argonaute-2 (AGO2) selectively ejects the passenger strand of the siRNA duplex. The retained guide strand directs RISC to recognize target viral RNA through perfect base-pairing complementarity, and AGO2 within the RISC subsequently cleaves and degrades the target viral RNAs(Hutvagner & Simard, 2008).

As the key effector component in the RNAi pathway, the AGO protein family plays a pivotal role in RNAi-mediated antiviral defense. In human and other mammals, multiple AGO isoforms are encoded, among which only AGO2 possesses RNase H-like endonuclease activity (Liu et al., 2004). However, recent studies demonstrate that AGO3 also retains cleavage activity and can cleave target RNAs under specific conditions. AGO3-mediated substrate cleavage depends on guide RNA specificity, though the precise mechanism remains elusive (Park et al., 2017). Generally, AGO proteins are composed of four conserved domains: the N-terminal (N) domain, the PAZ domain, the Middle (MID) domain and the PIWI domain. The MID domain recognizes and binds the 5′-phosphate group of small RNAs, while the PAZ domain anchors the 3′-hydroxyl group, synergistically stabilizing small RNAs(Ma, 2005; Parker et al., 2005). The MID domain also interacts with the seed region (nucleotides 2–8) of the small RNA, guiding complementary pairing with target RNA (Schirle et al., 2015). The N domain acts as a wedge to facilitate the unwinding of the small RNA duplex, leading to passenger strand release and inducing conformational changes in AGO that transition the RISC from a loading state to an active, slicing-competent state (Kwak & Tomari, 2012). In catalytically active AGOs, such as mammalian AGO2 and *Drosophila* AGO2, the PIWI domain contains an RNase H-like fold that cleaves target RNA fully complementary to the guide strand (Meister et al., 2004; Parker et al., 2004). Additionally, two linker regions-L1 and L2 respectively between the N and PAZ domains and between the PAZ and MID domains play critical roles in stabilizing the structure of the RISC(Yuan et al., 2005).

The production of vsiRNAs through the Dicer-AGO2 axis, which specifically cleave viral genomes, represents key evidence for the activation of RNAi-mediated antiviral immunity. Recently, Wu et al. demonstrated that wild-type coronavirus (Infectious bronchitis virus, IBV) and influenza virus (Avian influenza virus, AIV) can trigger antiviral RNAi and produce virus-derived siRNAs. The authors also constructed a vsiRNAs database for multiple animal (livestock/poultry) viruses and identified a position-dependent 3 bp motif named RWM that promotes Dicer processing within these vsiRNAs. Experimental validation confirmed enhanced Dicer cleavage efficiency to RWM-containing vsiRNAs. Significantly, synthetic vsiRNAs designed with RWM motifs exhibited antiviral activity in vitro and in vivo. This study establishes an RNAi-based antiviral strategy that directly targets conserved viral genomic regions, delivering a rapid screening approach, enhanced biosafety, and inherent capacity to break through host immune constraints while effectively avoid mutation risks associated with viral immune escape, thus providing an applicable strategy for advancing RNAi immunotherapy study (Wu et al., 2025).

Through long-term evolutionary arms races, viruses have evolved viral suppressors of RNAi (VSRs) to counteract the RNAi pathway and evade host immune pressure (Bivalkar-Mehla et al., 2011; de Vries & Berkhout, 2008; Voinnet et al., 1999). We will discuss VSRs in later parts.

### 2.2 Antiviral RNAi in plants and invertebrates

RNAi-based antiviral defense was first discovered in plants and serves as the primary antiviral mechanism (Jin et al., 2022). Plants employ intracellular Dicer-like protein (DCL) to sense and cleave viral dsRNAs, generating abundant primary vsiRNAs, which form the foundation of effective antiviral defense (Brodersen & Voinnet, 2006). Host RNA-dependent RNA polymerases (RdRPs) in plants exponentially amplify large amounts of viral dsRNAs, which further serve as substrates for DCL, being processed to produce new secondary vsiRNAs(Borges & Martienssen, 2015). Both vsiRNAs and secondary vsiRNAs target complementary viral RNAs, forming the RISC complex to mediate the degradation of viral genomes. In nearly all plant viruses, particularly pathogenic ones, VSRs are widely present. These VSRs achieve successful host invasion and promote viral propagation by directly or indirectly targeting key RNAi pathway factors, dsRNAs and vsiRNAs, as well as RdRPs critical for secondary vsiRNA biogenesis, thereby suppressing RNAi (Jin et al., 2022). Significantly, hosts have evolved countermeasures targeting VSRs directly to combat viruses’ evasion of antiviral RNAi. For instance, Zhu et al. discovered that PRMT6, a key regulator in plant immunity, suppresses the VSR activity of Tomato bushy stunt virus (TBSV) P19 protein by catalyzing its arginine methylation, thereby blocking P19-siRNA binding. Thus, while investigating VSR mechanisms remains critical, screening host-encoded antiviral proteins that antagonize VSRs not only provides innovative strategies for antiviral therapeutics but also establishes the molecular foundation for breeding naturally virus-resistant crops in plant systems (Zhu et al., 2024).

RNAi is also recognized as a critical antiviral immune pathway in invertebrates, with evidence suggesting that its efficiency correlates closely with viral infection prevalence (Obbard et al., 2006; Zhu & Palli, 2020). Taking *Drosophila melanogaster*—a well-studied model organism—as an example, RNAi plays a pivotal role in antiviral immunity of insects. Analogous to plant RNAi-based antiviral mechanisms, upon viral infection, Dcr-2 in *Drosophila* recognizes virus-derived dsRNAs and processes it into vsiRNAs. These vsiRNAs are subsequently incorporated into the RISC, where AGO2 mediates cleavage and degradation of complementary viral RNAs(Swevers et al., 2018). Studies show that *Drosophila* with mutations in Dcr-2 or AGO2 exhibit heightened susceptibility to RNA virus infections, with significant viral RNA accumulation detected in infected individuals (Galiana-Arnoux et al., 2006; van Rij et al., 2006). Recent research further reveals that even DNA viruses can trigger host RNAi-mediated antiviral defenses. For instance, vsiRNAs have been detected in *Drosophila* infected with Invertebrate Iridescent virus 6 (IIV-6) and in Helicoverpa armigera larvae infected with Helicoverpa armigera single nucleopolyhedrovirus (HaSNPV), providing experimental evidence that DNA viruses also activate RNAi-based immunity (Bronkhorst et al., 2012; Jayachandran et al., 2012). The invading DNA from the DNA virus-IIV-6-can also be sensed by the host non-canonical RNA polymerase Ⅱ (RNAPⅡ) and generate virus-derived dsRNA, which activates the antiviral siRNA pathway in *Drosophila* (de Faria et al., 2022). The activation of RNAi pathway by RNAPⅡ-derived dsRNAs is similar to the mechanism that mammalian RNA Pol III catalyzes the production of dsRNAs to trigger RIG-I sensing (Ablasser et al., 2009). Intriguingly, multiple insect viruses encode VSRs that specifically target RNAi pathway components to achieve immune evasion. Collectively, these findings underscore the indispensable role of the RNAi pathway as a vital antiviral defense mechanism in both plants and insects.

### 2.3 Controversies and advances in RNAi-mediated antiviral immunity in mammals

As previously mentioned, a key feature of RNAi as a core antiviral pathway in plants and invertebrates is the enrichment of vsiRNAs upon viral infection. However, earlier studies revealed that vsiRNAs are nearly undetectable in many differentiated mammalian somatic cell lines after viral infection, casting doubt on RNAi's antiviral role in mammals (Pfeffer et al., 2005). In mammals, viral infection triggers rapid recognition of viral dsRNAs by PRRs such as RIG-I-like receptors (RLRs) and Toll-like receptors (TLRs), initiating cascades that induce massive interferon (IFN) secretion (Gürtler & Bowie, 2013). These IFNs act on IFN receptors (IFNRs), activating downstream interferon-stimulated genes (ISGs) whose protein products play critical antiviral roles. For instance, RNA-dependent protein kinase (PKR) inhibits viral translation by phosphorylating the α subunit of translation initiation factor 2 (eIF2α)(Pindel & Sadler, 2011). Another example is that the 2′,5′-oligoadenylate synthetase (2′-5′OAS) activates another ISG, RNase L, to non-specifically degrade viral RNAs via oligoadenylate production (Chakrabarti et al., 2011). Furthermore, as the member of ISGs, ADAR1 modulates the function of dsRNA by directly catalyzing adenosine-to-inosine (A-to-I) RNA editing within dsRNA (Bass, 2002). The existence of such a multi-pathway, high-efficiency antiviral network has solidified the IFN system as the major antiviral mechanism in mammals.

Moreover, recent studies indicate that the IFN pathway has some suppressive role on antiviral RNAi. The ISG LGP2 in mammals binds Dicer to inhibit dsRNAs processing into siRNAs, thereby inhibiting RNAi. In cells lacking LGP2 or IFN signaling, dsRNA can trigger RNAi responses (van der Veen et al., 2018).

In the recent decade, emerging evidence demonstrates that RNAi does retain a non-negligible antiviral activity in mammals. For instance, in certain undifferentiated mammalian cells, such as human neural progenitor cells (hNPCs)(Xu et al., 2019) and mouse embryonic stem cells (mESCs)(Wianny & Zernicka-Goetz, 2000), following viral infections, long dsRNAs can activate RNAi and produce abundant vsiRNAs(Maillard et al., 2013). Consistently, researches from different groups have identified an oocyte-specific Dicer isoform (Dicer^O^)(Kennedy et al., 2015) or an antiviral Dicer (aviD)(Poirier et al., 2021), which lacks the N-terminal DExD helicase domain, exhibits higher dsRNA cleavage activity than full-length Dicer in undifferentiated mouse and human cells, as well as in organs from pre-weaning or adult mice. Overexpression of this N-terminally truncated human Dicer mutant in virus-infected 293T cells also generates vsiRNAs. Notably, aviD has been proved to produce more siRNAs from dsRNAs than Dicer and be more resistant to LGP2. In addition, the ability of aviD to process precursor miRNA (pre-miRNA) remains unaltered, which suggests that the aviD mutant may represent an evolutionarily retained adaptation in mammalian Dicer, likely preserved throughout evolution to combat viral invasion. Moreover, it has been reported that when viruses become defective in their VSR activities, these viruses can trigger the production of abundant vsiRNAs in infected mammalian somatic cells and in animals (Cullen, 2017). Furthermore, infection by viruses such as alphaviruses and coronaviruses have been found to yield readily detectable and functional vsiRNAs in differentiated somatic cells and in vivo (Kong et al., 2023; Wu et al., 2025). These findings collectively demonstrate that mammalian Dicer does retain the capability to cleave viral dsRNAs and RNAi does function as an antiviral defense in mammals in different physiological scenarios.

Although recent researches revealed the detection of low-abundance vsiRNAs in infected mammals, knowledge gaps persist regarding how host distributes viral dsRNAs to activate RNAi or IFN pathways-a critical question compounded by their inherent antagonistic crosstalk. Conversely, viruses have evolved VSRs to suppress hosts’ antiviral capacity to recognize and cleave viral dsRNAs into vsiRNAs. Delineating the molecular mechanisms by which VSRs antagonize host RNAi will deepen our understanding of dynamic host-virus interactions in antiviral RNAi and provide novel mechanistic foundations for antiviral therapeutic development. Hereafter we will summarize and exemplify various molecular mechanisms of VSRs.

## 3 Viral counteraction to RNAi

### 3.1 Strategies and multiplicity of VSR

#### 3.1.1 Binding and sequestering nucleic acid substrates

**Fig. 1:**
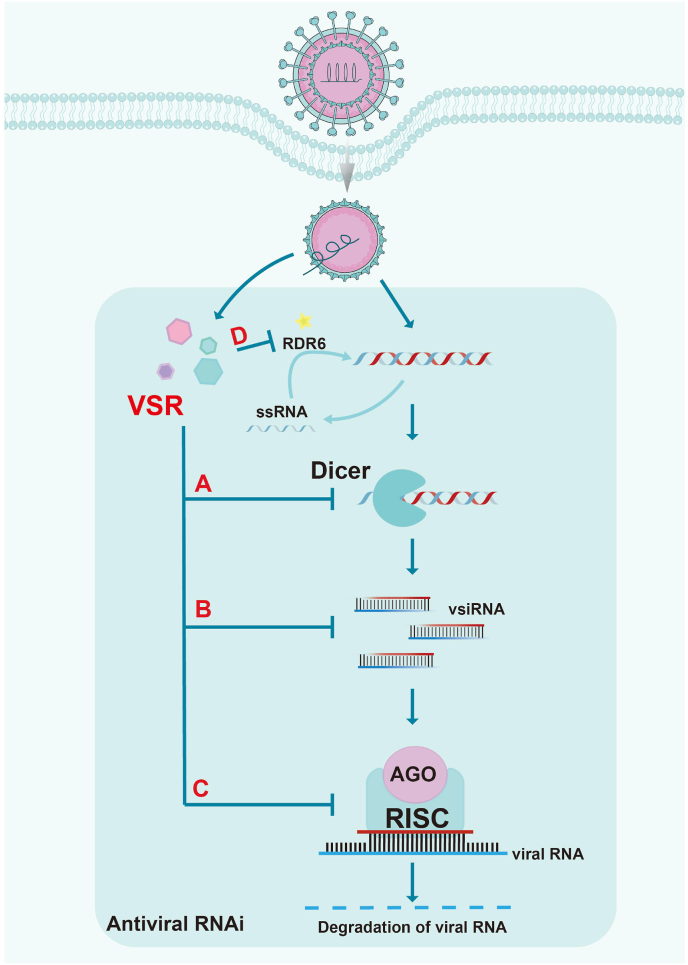
**Mechanism of antiviral RNAi immune pathway.** The inhibitory arrow denotes four well-established molecular mechanisms of VSRs-mediated suppression targeting the antiviral RNAi pathway, including: targeting Dicer; targeting vRI-dsRNAs or vsiRNAs; targeting AGO; targeting RNA-dependent RNA polymerases.

Diverse viruses encode their own VSRs to evade antiviral RNAi in distinct strategies. Among these approaches, certain VSRs act on RNAi intermediates and functional products, including vRI-dsRNA and vsiRNA, thereby impairing RNAi-mediated antiviral defenses. A classic example involves the B2 proteins encoded by various alphanodaviruses (*Nodaviridae*), which inhibit RNAi by directly binding long vRI-dsRNAs to block its processing by Dcr-2 into vsiRNAs, and also by sequestering vsiRNAs to prevent their loading into RISC (Fig. 1). This RNAi-suppressive activity of B2 is conserved across diverse alphanodaviruses, including the insect-infecting Flock House virus (FHV) and Wuhan nodavirus (WhNV), as well as Nodamura virus (NoV), which infects both insects and mammals (Lu et al., 2005; Qi et al., 2011, 2012; Sullivan & Ganem, 2005). Notably, B2 proteins from fish-infecting betanodaviruses, a distinct subfamily of *Nodaviridae*, exhibit similar RNAi antagonism despite significant sequence divergence from alphanodaviruses. For instance, the B2 protein of Greasy grouper nervous necrosis virus (GGNNV) binds dsRNA to suppress RNAi (Fenner et al., 2006) (Fig. 1A). Additionally, the P19 protein encoded by the plant virus TBSV forms a homodimer that specifically sequesters vsiRNAs, preventing their loading into the RISC complex (Silhavy et al., 2002) (Fig. 1B). Interestingly, most VSRs encoded by mammalian viruses, such as Enterovirus nonstructural protein 3A, Flavivirus nonstructural protein NS2A, Influenza A virus NS1, Coronavirus nucleocapsid (NP), Rubella virus capsid protein, Alphavirus capsid protein, Hepacivirus NS2, exert their VSR activities via binding to vRI-dsRNAs(Li et al., 2017; Qian et al., 2020; Qiu et al., 2017, 2020; Wu et al., 2025; Xu et al., 2021; Zhou et al., 2020) (Fig. 1A).

#### 3.1.2 Directly inhibiting the core enzymes of RNAi pathway

In addition to acting on vRI-dsRNAs or vsiRNAs, VSRs can directly target key proteins of the host RNAi pathway, such as Dicer and AGO, suppressing their activity or degrading them via a ubiquitination pathway to reduce RNAi efficiency. For example, in addition to its dsRNA/siRNA-binding activity, WhNV-encoded B2 directly binds to the RNase III and PAZ domains of Dcr-2 via its C-terminal region, thereby blocking the activities of Dcr-2 in processing dsRNA and incorporating vsiRNA into the RISC(Qi et al., 2012) (Fig. 1A). The Rotavirus (RV) encoded-nonstructural protein 1 (NSP1) serves as a VSR by triggering degradation of AGO2 via ubiquitin-proteasome pathway, resulting in inhibition of RNAi (Mukhopadhyay et al., 2019) (Fig. 1C). Moreover, during plant infection, vsiRNAs can transfer to host cells, where they serve as templates for the synthesis of new dsRNAs catalyzed by RDRs. These dsRNAs are further processed by the RNAi machinery into secondary vsiRNAs, which systemically spread to establish a whole-plant defense network, protecting newly developing tissues from viral invasion. In counteraction to this, the Rice yellow stunt rhabdovirus (RYSV) protein 6 suppresses secondary vsiRNAs biogenesis by targeting RDR6 (Guo et al., 2013), thereby antagonizing this host immune response (Fig. 1D). This inhibition not only blocks RNAi-mediated degradation of viral genomes but also disrupts the biogenesis of vsiRNAs, thereby promoting immune evasion.

#### 3.1.3 Multi-target synergistic inhibition

Numerous viruses achieve RNAi suppression through multi-target synergistic inhibition. Certain VSRs act simultaneously on critical proteins and nucleic acid substrates at distinct nodes of the RNAi pathway. For example, the 2b protein of Cucumber mosaic virus (CMV) not only binds dsRNA or siRNA to inhibit siRNA biogenesis and RISC loading but also directly interacts with AGO1, specifically blocking its slicer activity and thereby disrupting miRNA-mediated gene regulation (Goto et al., 2007) (Fig. 1A–C). Another example is the aforementioned WhNV B2, which exerts its VSR activity by binding to both vRI-dsRNAs/vsiRNAs and Dcr-2 (Qi et al., 2012) (Fig. 1A–B). In addition, some viruses leverage multiple factors generated during replication to collectively target RNAi pathways, synergistically counteracting antiviral RNAi. Notably, apart from virally encoded proteins, recent studies reveal that non-coding RNAs generated during viral replication also function in counteracting host RNAi-mediated antiviral immunity. For instance, Zika virus (ZIKV) employs multiple mechanisms to suppress RNAi: the viral NS2A protein directly binds vRI-dsRNA to prevent Dicer processing, while the capsid protein binds Dicer, interfering with vsiRNA production (Qiu et al., 2020; Zeng et al., 2020) (Fig. 1A). Moreover, subgenomic flaviviral RNA (sfRNA) produced in ZIKV-infected hNPCs competitively binds to RNA Helicase A (RHA) and Protein Activator of PKR (PACT)—two RNA-binding proteins associated with the RISC complex—thereby blocking their interaction with siRNAs and suppressing RNAi (Chen, Li, et al., 2024) (Fig. 1B). Dengue virus (DENV) expresses sfRNA to suppress RNAi, and its NS2A protein also binds vRI-dsRNAs, further underscoring the importance of evading antiviral RNAi (Schnettler et al., 2012) (Fig. 1A).

#### 3.1.4 Integration of pleiotropic functions

Generally, viral proteins with RNAi-suppressive activity often function as multifunctional effectors. These factors either participate in viral life cycle processes-such as replication, assembly, or trafficking-to enhance viral proliferation, or modulate host pathways to create a replication-favorable environment while enabling immune evasion. For instance, the SARS-CoV-2-encoded nucleocapsid (N) protein binds and sequesters dsRNAs to block Dicer-dependent vsiRNA production, effectively suppressing antiviral RNAi (Mu, Xu, et al., 2020) (Fig. 1A). Additionally, the SARS-CoV-2 N protein inhibits G3BP1-mediated stress granule formation, thereby disrupting RIG-I-dependent interferon signaling and further facilitating viral immune evasion and replication (Liu et al., 2022). Here we summarize several mechanisms by which VSRs in mammalian antagonize host RNAi, along with illustrative examples (Table 1) (see Table 2).

**Table 1:** Functionally identified VSRs of Mammalian RNA viruses.

Virus	VSR	Mechanism	Reference
Coxsackievirus B3	3A	Bind to vRI-dsRNAs and siRNAs	Mu, Zhang, et al. (2020)
Chikungunya virus	nsP2; nsP3	Function downstream of RISC loading	Mathur et al. (2016)
Dengue virus 2	NS2A	Bind to vRI-dsRNAs	Qiu et al. (2020)
Dengue virus 1-4	NS4B	inhibit Dicer activity	Kakumani et al. (2013)
Ebola virus	VP30; VP35; VP40	Bind to Dicer; Bind to TRBP-PACT complex	Fabozzi et al. (2011)
Enterovirus-A71	3A	Bind to vRI-dsRNAs	Qiu et al. (2017)
Hepatitis C virus	Capsid; NS2	Bind to vRI-dsRNAs	Zhou et al. (2020)
HIV-1	Tat	inhibit Dicer activity	Bennasser et al. (2005)
Influenza A virus	NS1	Bind to vRI-dsRNAs and siRNAs	de Vries et al. (2009)
Marburg virus	VP35	Bind to vRI-dsRNAs	Li et al. (2017)
Nodamura virus	B2	Bind to vRI-dsRNAs	Sullivan and Ganem (2005)
Rotavirus	NS1	Degrade AGO2	Mukhopadhyay et al. (2019)
Rubella virus	Capsid	Bind to vRI-dsRNAs	Xu et al. (2021)
SARS-CoV-1/2	Nucleocapsid	Bind to vRI-dsRNAs	Wu et al. (2025)
Semliki Forest virus	Capsid	Bind to vRI-dsRNAs	Qian et al. (2020)
Yellow Fever virus	Capsid	Bind to vRI-dsRNAs	Samuel et al. (2016)
Zika virus	Capsid	Bind to vRI-dsRNAs	Zeng et al. (2020)

**Table 2:** List of Abbreviations.

Abbreviation	Full Term
RNAi	RNA interference
PTGS	post-transcriptional gene silencing
siRNA	small interfering RNA
vsiRNA	viral small interfering RNA
RISC	RNA-induced silencing complex
VSR	viral suppressor of RNAi
AGO	Argonaute
TGS	transcriptional-level gene silencing
PEV	position effect variegation
miRNA	microRNA
dsRNA	double-stranded RNA
vRI-dsRNA	double-stranded RNA viral replicative intermediates
PAMP	pathogen-associated molecular pattern
PRR	pattern recognition receptor
HEL domain	N-terminal helicase domain
DUF	domain of unknown function
PAZ domain	PIWI-AGO-Zwille domain
dsRBD	dsRNA-binding domain
Dcr-2	Dicer-2
Argonaute-2	AGO2
N domain	N-terminal domain
MID domain	Middle domain
IBV	Infectious bronchitis virus
AIV	Avian influenza virus
DCL	Dicer-like protein
RdRP	RNA-dependent RNA polymerase
IIV-6	Invertebrate Iridescent virus 6
HaSNPV	Helicoverpa armigera single nucleopolyhedrovirus
RNAPⅡ	RNA polymerase Ⅱ
RLR	RIG-I-like receptor
TLR	Toll-like receptor
IFN	interferon
IFNR	interferon receptor
ISG	interferon-stimulated gene
PKR	RNA-dependent protein kinase
eIF2α	α subunit of translation initiation factor 2
2′-5′OAS	2′,5′-oligoadenylate synthetase
hNPC	human neural progenitor cell
mESC	mouse embryonic stem cell
Dicer^O^	oocyte-specific Dicer isoform
aviD	antiviral Dicer
pre-miRNA	precursor miRNA
FHV	Flock House virus
WhNV	Wuhan nodavirus
NoV	Nodamura virus
GGNNV	greasy grouper nervous necrosis virus
TBSV	Tomato bushy stunt virus
RV	rotavirus
NSP1	nonstructural protein 1
RYSV	Rice yellow stunt rhabdovirus
CMV	Cucumber mosaic virus
ZIKV	Zika virus
sfRNA	subgenomic flaviviral RNA
RHA	RNA Helicase A
PACT	Protein Activator of PKR
DENV	Dengue virus
TBV	Tombus virus
TCV	Turnip crinkle virus
CHIKV	Chikungunya virus
lncRNA	long non-coding RNA
AMP	antimicrobial peptide
CDK12	cyclin-dependent kinase 12
VTP	VSR-targeting peptide
CP	cyclic peptide
OPV	oral poliovirus vaccine

### 3.2 The role of VSR in virus pathogenicity

VSRs enhance viral pathogenicity by directly or indirectly inhibiting the host RNAi pathway, thereby improving viral replication efficiency, transmission capacity, immune evasion. For instance, CMV causes disease in tobacco, but CMV mutants with a defective VSR (2B) exhibit significantly attenuated virulence, resulting in reduced viral accumulation in infected plants (Kobori et al., 2005). Notably, 2B-deficient CMV mutants fail to establish effective infection in tobacco tissues, providing direct evidence for the critical role of VSRs in viral pathogenicity (Sunpapao et al., 2009). Similar phenomena have been observed in cells or animals infected by VSR-deficient viruses, including nodaviruses, enteroviruses, flaviviruses, coronaviruses, etc.

Beyond directly modulating viral replication, VSRs can hijack host miRNA pathways to dysregulate host factor expression and weaken antiviral defenses. The P19 protein encoded by Tombus virus (TBV), for instance, not only acts as VSR by sequestering vsiRNAs but also induces the expression of miR-168, an endogenous regulator of AGO1 mRNA, thereby suppressing AGO1 protein translation and its antiviral functions (Várallyay et al., 2014). Similarly, small RNA sequencing of DENV-infected mammalian cells revealed that the DENV NS3 protein can downregulate the abundance of specific endogenous miRNAs that target host mRNAs involved in DENV replication. Although the precise mechanism remains unclear, this process facilitates enhanced viral replication (Kakumani et al., 2015).

### 3.3 The role of VSR in virus-host co-evolution

The discovery of VSRs in eukaryotes-infected viruses, along with the elucidation of their mechanisms in modulating RNAi, has deepened our understanding of viral strategies to evade host immunity. Viruses adapt to host immune evolution through functional diversification of VSRs. Over long-term evolution, some viruses encode multiple effectors targeting distinct nodes of the RNAi pathway, which synergistically antagonize host RNAi-mediated antiviral defenses to achieve efficient immune escape. Typically, these RNAi-suppressing factors are evolutionarily conserved with the same genus. A prime example is that the N-terminus of Turnip crinkle virus (TCV) capsid protein P38 contains a GW motif highly conserved within the *Carmovirus* genus. This motif mimics host GW/WG repeat proteins that interact with AGO to facilitate RISC assembly, enabling P38 to directly and specifically bind Arabidopsis AGO1 and antagonize RNAi. Mutation of the GW residues substantially attenuates TCV virulence in host plants (Jin & Zhu, 2010). Similarly, the HIV-1 accessory protein Nef binds to AGO2 via its GW motifs and functions as a VSR of RNAi (Aqil et al., 2013). These GW-motifs, prevalent in diverse viral VSRs, competitively bind RISC components, destabilizing the balance between RNAi-based host defense and viral counter-defense. Besides, Chikungunya virus (CHIKV) nsP2 and nsP3 were identified as RNA-binding motifs with VSR activity that are highly conserved across the *Alphavirus* genus (Mathur et al., 2016). This dynamic process drives the co-evolution of viruses and their hosts.

From an evolutionary perspective, viral mutants encoding stronger VSRs gain a selective advantage by evading host RNAi surveillance, enabling them to outcompete ancestral strains. Conversely, the intricate immune evasion traits acquired through viral evolution drive host genome to evolve countermeasures that modulate RNAi activity. Zhang et al. discovered that the VSR of DCV-1A can bind the endogenous long non-coding RNA (lncRNA) VINR and enhance accumulation of VINR. The highly enriched VINR prevents Cactin degrading by ubiquitin-proteasome pathway. Consequently, Cactin stabilization triggers expression of antimicrobial peptides (AMPs), thereby activating a noncanonical innate immunity to defend against both the VSR-encoding virus and bacteria. This mechanism exemplifies host evolutionary adaptation to viral VSR-mediated antagonism of antiviral RNAi (Zhang et al., 2020). Another intriguing fact is that the host factor cyclin-dependent kinase 12 (CDK12) can interact with the VSR of FHV-B2-to affect its dimerization and inhibit its interaction with dsRNA, thereby abrogating RNAi suppression exerted by B2 (Zhang et al., 2025). This discovery indicates a host's capability to antagonize the VSR activity mediated by virus protein, which is a counter-counter-defense mechanism. In essence, the ongoing arms race between VSRs and host antiviral RNAi reflects a process of mutual adaptation and coevolution. As products of this evolutionary struggle, VSRs not only enhance viral immune evasion but also shape viral transmission patterns and host adaptability. Understanding VSR mechanisms provides critical insights into viral evolutionary dynamics, identifies novel antiviral targets, and offers a framework for studying host regulation of RNAi-mediated antiviral immunity.

## 4 Researches on VSR-targeting antiviral drugs and vaccines

### 4.1 Strategies and advancement

#### 4.1.1 VSR-targeting antiviral drugs

**Fig. 2:**
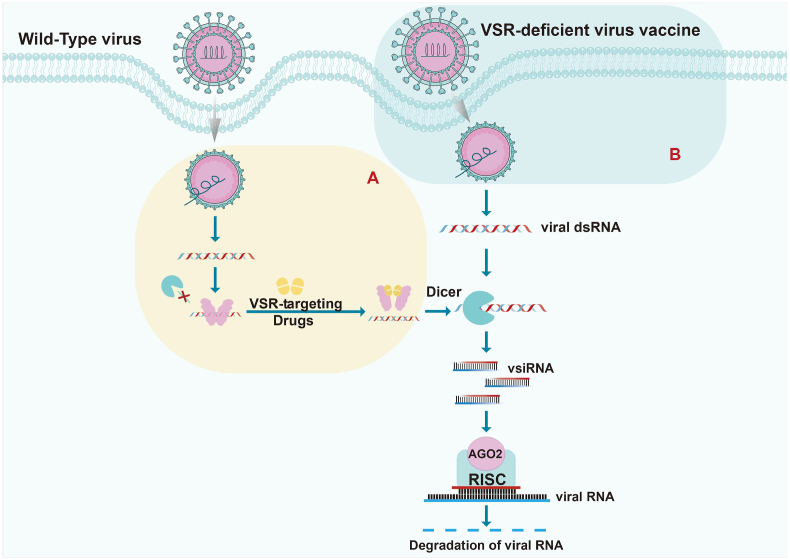
**TwoVSR-targetedantiviral strategies.** The yellow box represents antiviral drugs designed to competitively bind VSRs by mimicking their homodimerization interface. The green box represents VSR-deficient live-attenuated vaccines. Both classes of VSR-targeted antiviral strategies alleviate VSR-imposed suppression of antiviral RNAi immunity, thereby conferring robust protection in individuals where viral infection induces an active RNAi response.

Despite advancements in siRNA-based antiviral therapeutics, their clinical application remains constrained by potential off-target effects and viral mutation-driven resistance. However, emerging insights into virus-host RNAi interactions have identified highly conserved VSRs as promising drug targets. A canonical mechanism involves VSRs forming homodimers to sequester viral dsRNA, thereby blocking Dicer-mediated processing into vsiRNAs and disabling RNAi-directed viral RNA degradation. Representative examples include EV-A71 3A protein, NoV B2, IAV NS1, and SARS-CoV-2 NP, all of which employ this strategy to antagonize host antiviral RNAi. Targeting the homodimerization interfaces of such VSRs offers a novel therapeutic approach. For instance, Fang et al. designed a VSR-targeting peptide (VTP) named ER-DRI against EV-A71 3A's homodimerization interface, which competitively disrupts 3A homodimerization and vRI-dsRNA binding, unlocking Dicer-mediated vRI-dsRNA recognition and processing and triggering robust vsiRNA production. In murine models, this VTP administration resulted in abundant vsiRNA production, suppressed EV-A71 replication across multiple organs, alleviated clinical symptoms, and rescued infected mice from lethality (Fang et al., 2021) (Fig. 2A). Using the similar strategy, Chen et al. designed the VTP named GL to target SARS-CoV-2 N protein, liberated RNAi-mediated defenses in both cellular and animal models, exhibiting broad-spectrum efficacy against diverse coronaviruses by reactivating vsiRNA biogenesis (Chen et al., 2025). These findings underscore the therapeutic potential of VSR-targeting strategies to unlock the antiviral potency of RNAi responses. By counteracting VSR-mediated immune evasion, such interventions not only circumvent limitations of siRNA medicines but also exploit the evolutionary conservation of VSRs within virus families, reducing the likelihood of resistance.

#### 4.1.2 VSR-deficient live-attenuated RNA virus vaccine

Recent advances in mammalian antiviral RNAi have revealed its critical role in antiviral innate immunity in mammals. Ding and colleagues proposed a novel vaccine strategy: VSR-deficient live-attenuated RNA viruses that elicit rapid, adaptive immunity-independent protection in neonates and immunocompromised hosts (Chen, Han, et al., 2024) (Fig. 2B). For instance, neonatal mice inoculated with the VSR-disabled NoV (NoVΔB2) were completely protected from lethal NoV challenge within two days. Remarkably, this protection extended to immunodeficient *Rag1*^−/−^ mice (lacking mature B and T lymphocytes) regardless of age, and the immunity was virus-specific and remained effective in adult *Rag1*^*−/−*^ mice even 90 days post a single-shot immunization. These findings underscore that RNAi-mediated antiviral immunity provides immediate and durable protection independent of adaptive immune responses, highlighting its indispensable role in safeguarding immunologically vulnerable populations, such as newborns and individuals with compromised adaptive immunity. By employing an “exploit-the-virus-against-itself” strategy, VSR-deficient live-attenuated vaccines ingeniously make good use of viral vulnerabilities to activate host antiviral RNAi. Given that the anti-RNAi mechanisms are conserved in numerous medically important viruses such as coronaviruses, enteroviruses, flaviviruses, etc., this approach provides a novel perspective for developing vaccines tailored to vulnerable populations and may fundamentally reshape the antiviral therapeutic landscape for neonates and immunocompromised patients in the future.

### 4.2 Foreground and challenges

The observation that VSR-functional domains are evolutionarily conserved across the virus within the same genus or family suggests that VSR-targeting antiviral drugs hold broad-spectrum antiviral potential. By specifically releasing host RNAi suppressed by VSRs, these drugs avoid negative effects such as immune hyperactivation. However, practical development faces challenges including toxicity risks and low drug delivery efficiency. For example, VTPs based on homodimerization interfaces require extensive screening and engineering to minimize potential toxicity associated with their amino acid composition and off-target effects. Future efforts should focus on: optimizing drug structures via AlphaFold-predicted VSR-Drug interfaces; employing AI-driven deep learning to screen candidates; maximizing targeting efficiency while mitigating potential clinical risks. Furthermore, VTPs therapeutics suffer from rapid protease degradation in vivo, leading to inherently low stability, short half-lives, and constrained administration routes. This limitation prompts exploration of naturally occurring cyclic peptides (CPs)-characterized by backbone cyclization via peptide bond formation between N- and C-termini. Such structural closure confers superior protease resistance and extended circulatory persistence compared to linear analogs. Besides, CPs exhibit inherent lipid membrane affinity and acquire cell-penetrating capability through strategic chemical modifications, enabling selective tissue targeting. Therefore, inspired by natural properties of CPs, synthetic cyclization of existing VTPs enhances their transmembrane permeability and metabolic stability, unlocking previously inaccessible oral bioavailability potential. Beyond chemical modifications that optimize peptide physiochemical properties, classic biomaterials, adjuvant components, and advances drug delivery systems, including liposomes, hydrogels, microparticulate carrier systems, and nanoparticulate carrier systems, can also significantly enhance the stability, membrane adsorption, and penetration capacity of VTPs therapeutics (Chen et al., 2022; Zhang et al., 2018).

The VSR-deficient attenuated vaccines exploit RNAi-mediated antiviral immunity, offering superior protective advantages-especially in immunocompromised individuals and neonates. However, clinical translation still faces several key limitations: it requires prerequisite characterization of VSRs and their molecular mechanisms. Besides, like other attenuated virus vaccine, the VSR-deficient virus vaccine may face the similar challenge of regaining virulence through compensatory mutations. An example is vaccine-derived poliovirus strain regained virulence to cause dozens of polio outbreaks. Therefore, deleting a large part of VSR gene or even the whole VSR, together with strict risk assessment, would be beneficial to generate VSR-deficient virus vaccine (Stern et al., 2017).

## 5 Conclusions and outlook

RNAi serves as a central antiviral pathway in fungi, plants and invertebrates. Mounting evidence from recent studies demonstrates that the RNAi pathway also functions as an antiviral defense in mammals. Notably, RNAi-mediated immunity plays a pivotal role in undifferentiated cells where IFN signaling remains undeveloped. In mammals, the RNAi and IFN pathways form a collaborative antiviral network through competitive recognition of dsRNA and cross-regulatory mechanisms. Therefore, future research should focus on elucidating the interplay between RNAi and IFN pathways during viral infection, defining the non-substitutable antiviral roles of mammalian RNAi in specific physiological contexts. Concurrently, to address challenges with VTPs (e.g., limited stability, poor oral bioavailability), VSR-targeting antivirals could also be developed in alternative forms, such as peptidomimetics, cyclic peptides, small molecules, or nucleoside analogs. Furthermore, resolving the evolutionary conservation of VSRs and their interaction interfaces with host RNAi pathways will accelerate the development of broad-spectrum antiviral agents.

## CRediT authorship contribution statement

**Bowen Zhang:** Writing – original draft. **Xi Zhou:** Writing – review & editing, Supervision, Investigation, Funding acquisition. **Yujie Ren:** Writing – review & editing, Supervision, Investigation, Funding acquisition.

## Declaration of competing interest

The authors declare that they have no known competing financial interests or personal relationships that could have appeared to influence the work reported in this paper.
